# Managing in the new normal: Positive management practices elicit higher goal attainment, goal commitment, and perceived task efficacy than traditional management practices in remote work settings. An experimental study

**DOI:** 10.3389/fpsyg.2022.914616

**Published:** 2022-10-06

**Authors:** Lucas Monzani, Guillermo Mateu, Pilar Ripoll, Eva Lira, José María Peiro

**Affiliations:** ^1^Ivey Business School, Western University, London, ON, Canada; ^2^Department of Accounting, University of Valencia, Valencia, Spain; ^3^University Bourgogne Franche-Comté, CEREN, EA 7477, Dijon, France; ^4^Departamento de Psicología Social, University of Valencia, Valencia, Spain; ^5^Departamento de Psicología y Sociología, University of Zaragoza, Zaragoza, Spain

**Keywords:** goal setting types, authentic leadership, contingent rewarding, goal attainment, goal commitment, perceived task efficacy, self-leadership

## Abstract

The COVID-19 global pandemic will likely change how organizations conduct business. For example, a white paper from McKinsey claims that flexible and remote work arrangements (e.g., “working-from-home”) will become increasingly frequent in the “new normal” that will follow the COVID-19 pandemic. Our work is motivated by the premise that in a post-pandemic workplace, traditional management practices like unilaterally assigning goals and displaying contingent rewarding behaviors will likely be replaced by positive management practices. In this context, positive management practices include allowing employees to self-set their goals and displaying authentic leadership behaviors while managing them. However, whether these positive management practices are more efficient in sustaining performance is unknown. Our study benchmarked positive management practices against traditional management practices in a remote work environment, using three individual performance metrics: goal attainment, goal commitment, and perceived task efficacy. In a panel laboratory experiment consisting of a baseline measurement and two work sessions, we randomly assigned participants to an authentic vs. transactional leadership condition (amateur actor recording) and one of three possible goal-setting types (assigned, self-set, “do-your-best”). Our results show that participants in the authentic leadership × self-set goals condition outperformed all other experimental conditions. Further, a *post hoc* analysis revealed a serial mediation effect of (a) goal attainment and (b) goal commitment at time 1 on perceived task efficacy reports at time 2.

## Introduction

The COVID-19 global pandemic will likely change how organizations conduct business. In a survey from the Pew Research Center, 787 out of 915 innovation experts (86%) declared that the evolution of digital life would continue to feature “both positives and negatives” in post-COVID work contexts.^1^ The report concluded that to address this macro-trend, organizations should embrace “tele-everything”, understood as the virtualization of everyday tasks. As work virtualizes further, middle and line managers can expect increasing demands for flexible, remote work arrangements from their employees. Similarly, a white paper from McKinsey claims that flexible, remote, work arrangements will become increasingly frequent in the next years. Thus, leaders should embrace rather than resist flexible and remote work (de Smet et al., 2021).

However, delivering on these new flexible, remote work arrangements will likely create new challenges for managers. From a manager’s perspective, a “work-from-home” arrangement implies relinquishing the ability to monitor direct reports closely and correct deviations from existing norms. Thus, in this “new normal,” managers will need to find new ways to ensure that their employees remain committed and fulfilling their everyday tasks efficiently. With this challenge in mind, we inquired whether traditional management practices would be effective or if different management practices are needed to sustain performance when working remotely.

To answer the above research question, the main objective of the present study is to benchmark how positive management practices perform against transaction-oriented (traditional) management practices in remote work contexts. We propose that in a remote work context, adopting an authentic leadership style and allowing employees to self-set their goals (Erez et al., 1990; Avolio et al., 2004; Gardner et al., 2005) will result in higher goal commitment, goal attainment, and perceived task efficacy than adopting a transactional leadership style and a directive goal setting (Locke and Latham, 1990; Bass and Avolio, 1994). Thus, the present study can also inform the study of work motivation in the “new normal.” More precisely, our study explores a somewhat neglected boundary condition of Goal Setting Theory (Locke and Latham, 1990, 2013), a well-established motivational theory. Whereas Locke and Latham’s (1990) Goal Setting Theory has been strongly supported in the “old normal,” revisiting the adequacy of its predictions is important as the societal context in which these predictions were made evolves.

Following extant research on Goal Setting Theory, we chose three key performance indicators that can be informative when benchmarking managerial practices. More precisely, we chose goal attainment, goal commitment, and perceived task efficacy as the focal outcomes of our study. These three outcomes capture task-related behaviors and attitudes reflecting individual performance that are likely generalize across face-to-face and remote work settings.

A goal is the object or aim of an action, for example, to attain a specific standard of proficiency, usually within a specified time limit (Locke and Latham, 2002). Goal setting, then, is a process by which a goal setter formalizes a performance expectation into a goal. Instead, goal attainment refers to the (positive) outcome of exerting persistent and directed effort to pursue a goal. Usually, goals are set before a task starts, and goal attainment is assessed after a task ends.

Goal commitment is “one’s attachment to or determination to reach a goal, regardless of its origin” (Locke et al., 1988, p. 24). In other words, goal commitment is an essential condition by which goal setting affects goal attainment and perceived task efficacy. As Locke et al. (1988) argue, “it is virtually axiomatic that if there is no commitment to goals, then goal setting does not work” (p. 23). These authors argue that goal commitment is related to but distinct from goal acceptance. Goal acceptance captures an individual’s initial agreement to pursue a goal, whereas goal commitment captures an individual’s overall attachment until the goal is attained (Locke et al., 1988).

Finally, we distinguish perceived task efficacy (as operationalized in the present study) from general self-efficacy, as two related but distinct constructs. General self-efficacy is an core self-evaluative construct that captures an individual’s confidence in one’s future performance across tasks and contexts (Bandura, 1982). General self-efficacy predicts the attainment of self-set goals when goal difficulty is held constant (Locke and Latham, 2002), but it does not capture individuals’ self-evaluations of performance *after* a specific task. Thus, we focus on perceived task efficacy, as adapted to the individual level from the study by Kuhn and Poole (2000) on virtual teams. As perceived task efficacy collects individuals’ evaluations about how they performed in a specific task, it fits better the objective of the present study than self-efficacy.

Given the importance of these three constructs for organizations working remotely, our work aims to identify antecedents of these three outcomes, focusing on managerial practices within a specific type of remote work setting. Prior studies identified transformational leadership and participative goal setting as antecedents of goal commitment (Klein et al., 2013), and to some extent, as distal antecedents of goal attainment (Latham et al., 1988; Kirkpatrick and Locke, 1996). However, the indirect effects that these antecedents might have on perceived task efficacy as mediated by goal attainment and goal commitment has not been studied comprehensibly.

Something similar occurs for goal setting practices in remote work contexts. Prior studies explored goal setting practices in a remote work contexts (Wegge et al., 2007; Haslam et al., 2009), yet few studies explored the joint effects of a goal setter’s characteristics (e.g., leaders’ behaviors) and goals setting practices (e.g., directed vs. self-set) on a remote work context. By simultaneously investigating these lacunae in a single study, our work has the potential to offers valuable insights to managers interested in preparing their firms for the post-pandemic work context.

To pursue the main objective of this study, we conducted an exploratory laboratory experiment simulating remote work context. We tested four possible leadership style × goal setting type combinations in a sample of psychology and labor relations students, with and without work experience. Although conducting research in organizational behavior in student samples have been criticized (Rad et al., 2018), we believe that sample is appropriate when exploring phenomena that will matter for managing entry-level and junior employees.

### Theoretical framework

Without doubt, Locke and Latham’s (1990) Goal Setting Theory has been the most influential work motivation theory to date. The core prediction of this theory is that setting clear, specific, but also challenging goals has a motivational effect on individuals that leads to higher performance in a task that when goals are unclear and unchallenging. Attaining goals, in turn, results in increased job satisfaction, and organizational commitment (Locke and Latham, 2002). This was also proved to be true for individual performance in economic games. For example, Corgnet et al. (2018) ran an experiment using different agency models, and reported that agents performed better in the presence of goal setting, even under weaker monetary incentives. As Locke and Latham (2013) evidenced, the predictions of goal setting theory have been supported in almost every possible context.

However, goal setting theory still has a boundary condition that has been somewhat neglected in prior studies, which is the effect of how goals are set has on individuals’ goal attainment. Prior studies on goal setting showed that an actor’s performance will vary if the goals are set unilaterally by an authority figure or if set in a participative manner (Erez and Arad, 1986; Erez and Earley, 1987). In the “old normal,” a series of joint experiments helped resolve the controversy that mixed findings regarding goal setting type created (Latham et al., 1988).

Latham et al. (1988) concluded that there were no motivational differences between participative goal setting and directive goal setting, when a rationale for an assigned goal was provided (“tell & sell” approach). Instead, participative goal setting provided a cognitive effect that facilitated information exchange among team members that enhanced the team’s strategies on how to better pursue their shared goal. This effect was also detected in remote work contexts (Wegge et al., 2007; Haslam et al., 2009). Latham et al.’s (1988) study omitted the self-setting of goals, that is, the individual-level version of participative goal setting (Locke et al., 1984). Thus, the literature is unclear if the cognitive advantage of participative goal setting on task performance also applies to self-set goals. A deeper examination of self-set goals is necessary to maximize remote workers’ task performance.

Allowing employees to self-set their goals seems as a more positive managerial alternative to assigning goals directly. By allowing employees to self-set their goals, managers empower employee to self-determine their task-oriented behaviors, rather externally regulating them with extrinsic rewards or punishments (Deci and Ryan, 2000). Moreover, it would seem impractical to use only monetary rewards to reward employees who self-set goals, as “rational” employees would just selfishly reduce their task goals to exert the minimum effort possible (Konow and Earley, 2008). For example, studies have shown that participants self-setting their goals without a monetary incentive outperformed participants self-setting their goals under a monetary incentive. This intrinsic motivational effect of self-set goals was stronger when goals were moderate or difficult (Erez et al., 1990; Herranz-Zarzoso and Sabater-Grande, 2018). Given that self-set goals elicit intrinsic motivation, we expect a main effect of self-set goals on our three outcomes when operation in remote work settings. Thus, we predict:

**Hypothesis 1:** In a remote work context, self-setting goals will result in higher (a) goal attainment, (b) goal commitment, and (c) perceived task efficacy than if a manager assigns goals.

#### Leadership styles

Yukl et al. (2002) argue that most leadership styles can be organized into three meta-categories, “task,” “relations,” and “change.” The “task” meta-category captures behaviors by which leaders ensure that followers accomplish their tasks goals. The “relations” meta-category collects behaviors aimed at improving how well leaders relate with followers. The “change” meta-category refers to behaviors that elicit transformative change. Yukl’s “task” and “relations” meta-categories allow to distinguish between traditional and positive managerial practices.

Yukl’s categorization complements the earlier work of the late Bass (1995). Bass argued that leaders tend to exert either transactional (e.g., rewards contingent of successful performance and reducing deviations from norms) or transformative influence (e.g., inspiring followers, teams and organizations to a better state of affairs; Bass, 1985, 1995). Yet, whereas transactional behaviors only energize followers to perform up to their leaders’ expectations (“task”), transformational behaviors would inspire followers, teams, and organizations to exceed their leader expectations and thus increase organizational performance (“relations” + “change”).

A myriad of studies and meta-analyses supported Bass’ core premise (Judge and Piccolo, 2004). However, with the turn of the century, a new consideration was necessary (Bass and Steidlmeier, 1999). The public and corporate scandals that culminated in the 2008 wall street crash revealed how many allegedly “transformational” leaders misused their charisma to influence followers to exceed their goals in tasks aimed at satisfying their leaders narcissistic needs, rather than protecting stakeholders shared interests. Bass and Steidlmeier (1999) denounced the self-serving use of leader influence as pseudo-transformational leadership (Barling et al., 2008; Christie et al., 2011). As a result, the study of transformational leadership was divided into *authentic* transformational and *pseudo*-transformational leadership, and then refined into authentic leadership theory (Avolio and Gardner, 2005; Gardner et al., 2005).

Authentic leadership theory claims that leaders’ can deliver sustainable veritable performance without necessarily engaging in a charismatic rhetoric (Avolio et al., 2004; Gardner et al., 2005). Through self-awareness and self-regulated behaviors, leaders elevate followers, teams and organizations (Gardner et al., 2005; Hernandez et al., 2011; Monzani and Van Dick, 2020). Authentic leadership theory describes leaders’ self-regulation invoking facets such as relational transparency, an internalized moral perspective, and a balanced processing of information. In turn, by being self-aware and self-regulating their behaviors authentic leaders become exemplary role models in their followers’ eyes. Extant systematic and meta-analytic reviews established the positive effect of authentic leadership on numerous individual and organizational outcomes (Gardner et al., 2012; Banks et al., 2016; Hoch et al., 2018).

Despite these encouraging results, there is a caveat worthy of note regarding the operationalization of authentic leadership (AL; Walumbwa et al., 2008). Two meta-analyses revealed an overlap between the existing measures for transformational and authentic leadership (Banks et al., 2016; Hoch et al., 2018). Whereas critics used these results to delegitimize authentic leadership theory (Alvesson and Einola, 2019; Alvesson, 2020), a more constructive lecture shows that these findings support Bass and Steidlmeier (1999) theorizing about transformational leadership being rooted on authenticity. These meta-analysis, however, show the importance of employing experimental designs and exogenous variables to ensure that authentic leadership research does not suffer from endogeneity bias (Antonakis et al., 2010).

In this regard, few studies benchmarked AL’s self-based influence mechanisms against leaders’ reinforcement-based influence mechanisms (contingent rewarding) in the same study (Monzani et al., 2015a). Whereas both transactional approaches and authentic-transformational styles are known to influence followers’ attitudes and behaviors, without a proper benchmark, scholars cannot determine *if* and *to which extent* authentic-transformational leadership might be *more* effective than transactional behaviors in eliciting followers’ outcomes (Judge and Piccolo, 2004). Disentangling self-based from charisma-based influence would allow determining the relative importance of self-based mechanisms in eliciting followers’ outcomes, and in this way, providing a meaningful contribution to the positive leadership literature.

Finally, our literature review shows that as it occurs with goal setting, most of authentic leadership research on remote work contexts was conducted at the team-level of analyses and using self-report scales. For example, authentic team leadership related to virtual teams’ performance through dyadic or group-level mediators, such as high-quality personal relations, information sharing (Hahm, 2017), or when task interdependence was high (Zhang et al., 2021). Again, there is little experimental evidence about individual-level influence of authentic leadership on followers operating in a remote work context. Given the scarcity of prior experimental research on the effect of authentic leadership on followers’ outcomes while working in remote work settings, we formulate the following exploratory hypothesis:

**Hypothesis 2:** In a remote work context, displaying authentic leadership behaviors will elicit higher (a) goal attainment, (b) goal commitment, and (c) perceived task efficacy in follower than displaying transactional leadership behaviors.

#### Interactive effects of goal setting types and leadership styles within remote work settings

In prior sections, we made a case for the importance of benchmarking management practices to determine which practice sustains employee performance in remote work settings (e.g., “working-from-home” arrangements). We also claimed that said managerial practices could be partially deconstructed as a function of goal setting types and leadership styles. Thus, after such decomposition, we can divide management practices into two categories, traditional management practices and positive management practices.

Traditional management practices capture what Yukl collected in the “task” meta-category and Bass characterized as reinforcement-based practices (Bass, 1995; Yukl et al., 2002). These management practices have been described as behavioral economics as the core elements by which traditional managers enforce their norms and policies (Zehnder et al., 2017). Therefore, if follows that a directive goal setting type has a strong conceptual and practical fit with a transactional leadership style. Thus, a stronger positive effect on goal outcomes should be expected than the individual effect of directive goal setting of transactional leadership behaviors.

An alternative approach to traditional management is grounded on positive leadership (Monzani and Van Dick, 2020). A positive management approach still acknowledges the importance of delivering profit to organizational shareholders, but also of doing so in socially and environmentally responsible way that considers the interest of other stakeholders (employees, clients, and society). This notion is an extension of stakeholder theory (Mitchell et al., 1997) and has been termed the “triple bottom line” in the managerial accounting literature (Elkington, 1998). Positive management practices share the same “ends” than traditional management practices but differs in the means by which such ends are pursued (and attained).

Because of their positive impact on their relations with their followers, authentic leadership behaviors would fall in the intersecting space between “relations” and “change” meta-category proposed by Yukl, as authentic leaders elevate followers through positive, growth-oriented social exchanges (Ilies et al., 2005; Monzani and Van Dick, 2020). The ability of authentic leadership of increasing followers’ self-determination by satisfying their psychological needs nuances our predictions regarding the impact of self-set goals on individual performance.

We claim that displaying an authentic leadership style in combination with allowing employees to self-set goals are core components of positive management practices, as they develop followers’ self-awareness and self-regulation (Gardner et al., 2005). Authentic leadership scholars argue that authentic leaders deliver sustainable and veritable organizational performance by increasing the psychological capital, commitment of their followers and the teams they lead (Avolio et al., 2004; Leroy et al., 2015). For example, at the team level, authentic leadership direct predicted team reflexivity and indirectly predicted a team’s performance (Lyubovnikova et al., 2017). This study aligns with the findings of Latham et al. (1988) which claimed a cognitive effect of participative goal setting on how teams strategize to purse their collective goals.

In the “old normal,” the extant evidence we reviewed suggests that at the team level, combining authentic leadership and participative goal setting would be a positive management practice that should increase a team’s goal attainment and effectiveness. Correlational findings suggest that is also the case for authentic leadership virtual team’s performance (Hahm, 2017), yet the role of goals were not considered in these and other correlational studies (Kahai et al., 2007). However, can we expect these team-level results translate to individuals when operating in remote work environments (e.g., work-from-home)? There are theorical and empirical arguments to expect so.

Theoretically, Ilies et al. (2005) proposed that authentic leaders and followers mutually influence each other growth-enhancing social exchanges. Authentic leaders elevate followers through exemplary role modeling, but their followers legitimize leaders by identifying with their leaders and incorporating their self-regulated behaviors into their core selves. Thus, it is plausible to expect that after a series of growth-oriented exchanges, followers might experience increased self-awareness and self-regulation, becoming more proficient in regulating their behavior while conducting their work. In other words, we claim that through these positive exchanges, authentic leaders enhances their followers’ self-leadership (Neck and Houghton, 2006). Self-leadership is a process of influencing and leading oneself in which individuals regulate their behavior through behavioral and cognitive strategies (self-rewarding, self-cueing, self-goal setting).

Recent studies show how self-leadership protected remote workers wellbeing during the COVID-19 pandemic. Müller and Niessen (2019) reported that part-time teleworkers self-set goals more often while working remotely than when working in the office, which increased their work satisfaction. Similarly, Costantini and Weintraub (2022) reported that self-setting goals and self-rewarding strategies elicited job crafting and increased work engagement when working from home. Finally, self-set goals predicted remote workers’ job meaningfulness and negatively predicted burnout (Sjöblom et al., 2022). Thus, matching this follower self-leadership behavior with a positive leadership style should increase followers’ individual performance and shape positive attitudes toward their efficacy when performing a task in a remote work setting.

Conversely, we anticipate that matching self-set goals with a transactional leader might be counter-productive to enhancing individual performance in remote work settings. This is because, in general, transactional leadership behaviors are more focused in limiting followers’ agency in favor of compliance, than on fulfilling followers’ need for autonomy (Ryan and Deci, 2000). Further, transactional leaders’ focus on reducing deviation from norms signals that external behavioral regulations are preferred to intrinsic regulations. Remote workers would limit themselves to fulfill external expectations instead of exceeding them. Thus, instead of increasing their followers’ perceived task autonomy, transactional leaders would shut-down any attempt to do things differently (or even better). Thus, we expect that combining a transactional leadership style and self-set goals should results in less goal attainment, commitment, and perceived task efficacy than any set of managerial practices. Therefore, we make the following predictions:

**Hypothesis 3:** In a remote work context, displaying authentic leadership behaviors and allowing individuals to self-set their task goals will result in higher (a) goal attainment, (b) goal commitment, and (c) perceived task efficacy in follower than displaying transactional leadership behaviors and assigning goals directly.

## Materials and methods

### Participants

Our sample consisted of two-hundred and forty part-time workers and students from a Spanish university. We discarded 26 participants due to a data recording error. The final sample consisted of 214 participants (67.76% female). At the time of the experiment, participants’ age ranged from 18 to 30 years old (*M* = 21.79; SD = 4.81). A total of 31.78% of our sample were employed (part-time jobs), 56.25% were in their first year, 37.5% were about to graduate, and 6.25% were postgraduate students. The dataset employed in the present study is stored at an online repository and publicly available at: https://osf.io/n9ty5/?view_only=13a08d2c96e64e73ae9440b0aa612e36.

### Materials

Participants were voluntarily recruited from the university, and they were endowed with 30% of their final marks for their participation in the present study. Moreover, participants could satisfy the course requirement by choosing their participation, or participants who did not desire to participate in the study could choose a class-related exercise (one student did).

### Design and procedure

After providing their informed consent, all the participants worked individually on a PC in a room with cubicles that accommodated 14 participants. The first author designed software and user interface that handled random assignment to conditions, experimental manipulations, work sessions, task feedback, and self-report questionnaires. All data were stored in a restricted folder within the university’s secure cloud server.

The experiment consisted of three parts: (T_0_) an initial baseline measurement and two work sessions (T_1_ and T_2_), with a 7 day separation period between each work session. The 7-day time frame was selected to accommodate participants and employ our laboratory more efficiently. After each work session, we administered post-session questionnaires. At the beginning of the first session, the software on each PC showed a welcome screen explaining the background story to our participants. Participants would play the role of a manger who reported directly to a CEO of a fictitious start-up and had to coordinate the efforts of six direct reports. Immediately after that, participants watched a 5-min video from the CEO, where an overarching distal goal was set (attaining 4 out of the 6 possible task goals in a work session). The content of the video varied according to our leadership style condition.

Each direct report presented participants with six problematic situations on the following six screens. Three of the six problematic situations would be resolved by conducting intellective tasks, and the remaining three would be solved by conducting a creative task. The intellective tasks had a demonstrably correct answer (Straus, 1999), and required participants to use cognitive skills, such as critical thinking, recognizing assumptions, or deductive reasoning, to understand a problem and reach a single solution. Instead, the goal of each generative task was to generate at least a minimum number of ideas in a specific period. Generative tasks also require cognitive skills but mainly draw on divergent and convergent thinking (Guilford, 1959). Divergent thinking involves approaching problems from different angles to produce as many alternative outputs as possible, whereas convergent thinking involves integrating these outputs into a coherent yet elegant gestalt (James and Asmus, 2001). See Supplementary Appendix I for a detailed experimental timeline and Supplementary Appendix II for a detailed description of each task.

After each of the six tasks within work sessions 1 and 2, a dynamic feedback system provided real-time feedback regarding participants’ performance. The dynamic feedback system was designed following Monzani et al. (2015a,b). More precisely, the real-time feedback screen consisted of a brief scripted commentary of the CEO about participants’ task performance, which varied according to (a) each leadership style and (b) participants’ actual past performance. Task feedback consisted of information on whether the task goal was attained (or not), plus the time required to complete the current task and the accumulated results of previous tasks. Third, procedural feedback was provided after every task (this consisted of general guidelines to enhance brainstorming for the generative task or an explanation of the correct answer on intellective tasks; see Supplementary Appendix III).

We manipulated the variable *goal-setting type* to obtain three conditions, two experimental (assigned and self-set) conditions and one control condition. In the control condition, goals were unspecific (“Do your best”). Instead, in the assigned condition, the CEO assigned goals were clear and specific but unilaterally. In the self-set condition, participants could self-set their goals, using the prior dynamic feedback to guide their decisions.

In the unspecific goals condition, participants were told to do their best to reach the overarching goal, and no information about specific task goals was displayed. Participants in the assigned goals condition were told before each task the expectations about their task outputs (how many ideas were required in generative tasks or how much time they had to solve the intellective task), without being able to allocate extra time to a task or decrease the number of expected ideas. In the self-set condition, participants could allocate more time to a single task at the expense of the overall time or increase their goal to obtain a higher score.

We manipulated leadership styles using a multimedia video. Unlike live actors, a multimedia video is a useful way to ensure that all participants receive the same stimuli within a condition (Shea and Howell, 1999). First, in the authentic leadership style condition, participants watched a male amateur actor giving a speech describing himself as a CEO who is highly self-aware, with a strong moral perspective, balanced information processing, and transparent in their work relationship (Walumbwa et al., 2008). In the contingent-rewarding condition, the same amateur actor stressed how rewards would only follow successful performance and encouraged participants not to deviate from the firm’s norms and policies (Podsakoff et al., 1990; see Supplementary Appendix III for detailed examples of our leadership manipulation script).

The intersection of these two factors results in a 2 × 3 inter-group design. Each participant was randomly assigned to one of the six resulting experimental conditions. In the authentic leader conditions, the unspecific goals condition had 39 participants, the assigned goals condition had 38 participants, and the guided self-set goals condition had 40 participants. In the contingent-rewarding leader conditions, the unspecific goals condition had 36 participants, the assigned goals condition had 39 participants, and the self-set goals condition had 36 participants.

### Measures

#### Goal attainment

Participants obtained a score ranging from 0 to 1 if they attained the goal of each task within each work session (six in all). If the goal was not achieved because a task was performed incorrectly, or participants ran out of time, they scored 0 points. For intellective tasks, if the goal (e.g., providing a correct answer in the specified time) was attained, a score of 1 point was applied. Two intellective tasks included achieving secondary goals, which allowed partial scoring (up to 12 partial units of 0.083 points each). On generative tasks, if individuals reached the goal of providing a requested number of ideas (or self-set by the participant), they obtained a score of 1 point. To obtain an overall goal attainment score, we aggregated the scores from each task to form a single score that ranged from “0” to “6.” For the unassigned (“Do your best”) condition, the software would consider the goal achieved if the participant fulfilled the same criteria as in the assigned goals condition; however, this criterion was not communicated to the participants in the unassigned goal-setting type condition.

Furthermore, because a strong goal commitment might encourage dishonest goal-related strategies (Schweitzer et al., 2004; Barsky, 2008), we conducted *ex post-facto* checks to ensure the reliability of our goal attainment measure. Whereas using a computer simulation minimized the possibility of dishonest behaviors on intellective tasks, our software could not perform a semantic analysis of the ideas generated in real-time. Hence, our generative tasks may have allowed dishonest task strategies, such as repeating (by using the example provided as a new idea or entering the same content in multiple ideas) or lying (by purposely suggesting an idea that does not fit the task parameters).

Two doctoral students rated almost 20% of the total number of ideas generated in the whole experiment (2,253 out of 11,711) to address such possibilities using a sub-routine of our software user inter-phase (UI). The UI randomly selected an equal number of ideas from each generative task. Ideas that intentionally deviated from the task instructions (lying) or paraphrased the same concept several times (repeating) were discarded. One of the raters evaluated the remaining 80% of ideas (9,458), eliminating dishonest ideas (repeating and lying), and adjusted each participant’s goal attainment scores for each generative task of both work sessions. An example of cheating in work session 1, task 1 (which required proposing ideas about why the fictitious company was a great place to work) would be “because this company offers very strong job security,” when the background story for the experiment clearly specified that the fictitious company was evaluating moving its production offshore to a Southeast Asian country. In turn, an example of repeating would be using the example provided in the user interface as another idea. For example, in work session 1, generative task 1, where participants had to propose sales pitches to encourage customers to buy a portable solar charger, the example provided was that it had a USB outlet. Thus, we scored the proposed idea as repeating if participants wrote something like “you can use the portable solar charger to charge your IPod.”

We relied on self-reports to measure participants’ goal commitment and perceived task efficacy. All questions were rated on 5-point Likert-type scales ranging from “1 = strongly disagree” to “5 = strongly agree.”

#### Goal commitment

We used five items from Hollenbeck et al.’s (1989) scale. Examples are “I am strongly committed to pursuing this goal” and, “I think this goal is a good goal to shoot for.” Cronbach’s α was 0.61 and 0.88 for work sessions 1 and 2, respectively.

#### Perceived task efficacy

We adapted four items from a scale developed by Burke et al. (2001). Sample items are “I considered and evaluated information and evidence related to the issues of today’s work session,” and “I considered an adequate number of alternative ideas.” Cronbach’s α was 0.79 and 0.81 for work sessions 1 and 2, respectively.

### Data analyses

We used multivariate analyses of covariance (MANCOVA) and Hayes’ PROCESS macro (Hayes and Preacher, 2014) as our main analytical procedures. More precisely, we relied on MANCOVAs to conduct preliminary checks on (a) demographic characteristics of our sample, (b) our leadership style manipulation, and (c) *post hoc* checks regarding participants’ dishonest behaviors. We used RM-MANCOVA to test hypotheses 1 and 2 and constructed multivariate regression models in IBM’s SPSS utilizing the PROCESS macro to test for indirect effects.

First, we explored the data of our experimental groups to ensure that there were no significant demographic differences after participants’ random assignment to conditions. To this end, we entered participants’ age, gender assigned at birth, and work experience respectively as dependent variables and our two exogenous variables (goal setting types and leadership styles) as fixed factors. Non-significant differences would suggest that the random assignment had effect.

Second, to conduct manipulation checks for our leadership manipulation, we entered authentic leadership dimensions as dependent variables in a MANCOVA and our leadership manipulation as a fixed factor (“0 = Contingent Rewarding”/“1 = Authentic”). We used the Spanish version of the Authentic Leadership Questionnaire (ALQ) to measure these four dimensions after each work session (Walumbwa et al., 2008; Moriano et al., 2011).

Whereas the ALQ has recently been under scrutiny due to its underlying conceptualization (Alvesson and Einola, 2019; Alvesson, 2020), additional psychometric evidence of this questionnaire’s validity has been provided (Avolio et al., 2018). All items we rated using a 5-point Likert scale with values ranging from: “1 = not at all” to “5 = frequently, if not always.” For work session 1, ALQ Cronbach’s alpha was α = 0.87 and α = 0.89 for work session 2. If our manipulation was effective, participants in the authentic leader condition should report significantly higher scores than those in the contingent-rewarding leader condition.

Third, we conducted repeated-measures multivariate analyses of Covariance (RM-MANCOVA) to test hypotheses 1 and 2. We entered goal attainment, goal commitment, and perceived task efficacy as dependent variables and goal-setting types (unspecific goals, assigned, and guided self-set goals) and leadership style (contingent rewarding vs. authentic) as fixed factors. We conducted pairwise comparisons by estimating marginal mean differences across conditions and applying Bonferroni’s correction. In multivariate models, estimated marginal mean differences (*I–J*) are equivalent to *post hoc* comparisons in analyses of variance (ANOVA). Further, we explored simple cell differences to clarify the interactive effect between goal-setting types and leadership styles. To this end, we conducted ANCOVAs for each cell of our dependent variables for work sessions 1 and 2.

### Common method bias

Common method bias has been identified as one of many sources of bias in social sciences (Podsakoff et al., 2012). A common method bias refers to the systematic variation that results from employing in a single study the same type of instrument or method (e.g., self-reports). Common method bias is problematic as it might influence the strength of relations observed when testing hypotheses, leading to misinterpretation of results. Whereas several recommendations to reduce common-method variance statistically exist (CMV; Richardson et al., 2009), other scholars argue that incorporating measures and instruments of different nature, that is, combining self-reports with exogenous instruments and observable data is a practical way to address CMV concerns (Podsakoff et al., 2003). We followed this recommendation by manipulating our predictor variables and using different methods at different stages of our regression models (observed variables for performance and self-reports for attitudinal criteria).

### Control variables

First, we controlled for followers’ attributions of charisma because, as mentioned above, we aim to explore differences between reinforcement-based and those positive leadership styles that do not rely on charisma as the main influence mechanism. Thus, we statistically controlled for followers’ attributions of the leader’s charisma by adjusting five items from Platow et al. (2006). Example items are: “My leader has a vision that spurs people on” and “My leader has a special gift for seeing what is worthwhile for others to consider.” Cronbach’s α = 0.85 for work session 1, and α = 0.88 for work session 2.

Second, given that the effect of goal setting types assumes equal goal difficulty across tasks, and prior studies show a relation between goal commitment and goal difficulty (Locke and Latham, 2002, 2013), we used subjective and objective indicators to control for goal difficulty. First, we asked participants to rate their overall perception of the effort required to complete the work session tasks by indicating their level of agreement with the item: “How much effort was required to complete these tasks?” using a 5-point Likert-type scale ranging from 1 = “Extremely Easy” to 5 = “Extremely difficult” (Kuhn and Poole, 2000). We programmed our software interface to measure the time that each participant needed to complete each task in each work session. In line with traditional studies in goal-setting research (Dossett et al., 1979), non-significant differences in time across tasks and conditions would indicate that the goal difficulty level was similar across the goal-setting type conditions. Similar goal difficulty across conditions would then enable making robust comparisons across goal setting type conditions.

Finally, we controlled for whether if our participants were early or “Gen Z” millennials. To do so, we first determined the year our participants were born by subtracting participants’ age (in years) from the date from the experiment. Having determined participants year of birth, we then created a dummy coded variable (0 = “Early Millennials” vs. 1 = “Gen Z Millennial”) and coded any participant born before or during 1994 as an early millennial. We chose 1994 as a cut-off criterion because 1994 was the year that the internet was “born” to the general public.

Whereas there are no generally accepted cut-off values between millennial generations, the emergence of the internet fundamentally shifted how “digital natives” relate to work. Any participant born after 1994 was classified as “Gen Z Millennials.” Given that our leadership style manipulation involved a millennial man acting as the CEO, we anticipate that a millennial leader might not have the same effect on same-age participants as younger participants. Finally, we controlled for potential differences between working and non-working millennials as well. To this end, we dummy coded “Employment status” as 0 = “Unemployed” and 1 = “Part-time employee” and entered them as a predictor in all our statistical models.

## Results

### Preliminary checks

Our manipulation checks revealed that our leadership manipulation had the intended effect, with no secondary effects on participants’ task-related behaviors (e.g., lying or repeating). Whereas the results of the first work session yielded no significant differences in the multivariate test [Wilks’ Λ = 0.99, *F*(4,220) = 0.80, n.s., partial η^2^ = 0.01], we found an effect of gender assigned at birth. Hence, we re-ran our analysis, splitting our sample by gender assigned at birth.

In line with prior theorizing (Eagly, 2005) and studies exploring gender assigned at birth and authentic leadership (Woolley et al., 2011; Monzani et al., 2015, 2021), significant differences in the expected direction between leadership style conditions were found for men [Wilks’ Λ = 0.82, *F*(4,66) = 3.51, *p* < 0.05, partial η^2^ = 0.17], but not for women [Wilks’ Λ = 0.98, *F*(4,148) = 0.65, n.s.]. For work session 2, estimated marginal means for all four authenticity dimensions were higher in the authentic leader condition for all participants, regardless of their gender assigned at birth [Wilks’ Λ = 0.96, *F*(4,220) = 2.465, *p* < 0.05, partial η^2^ = 0.04]. Hence, our leadership manipulation produced the intended effects for men in work session 1, and for all participants in work session 2.

#### Dishonest behaviors

We found significant differences between work sessions [Wilks’ Λ = 0.86, *F*(2,219) = 18.08, *p* < 0.001, partial η^2^ = 0.14] in repeating (*M* = 0.07, 95% CI [0.01, 0.14] for work session 1 vs. *M* = 0.12, 95% CI [0.08, 0.16] for work session 2). Similarly, we found differences in lying (*M* = 0.33, 95% CI [0.29, 0.38] for work session 1 vs. *M* = 0.47, 95% CI [0.42, 0.52] for work session 2). No differences emerged across goal-setting types [Wilks’ Λ = 0.99, *F*(4,438) = 0.44, n.s.], leadership style [Wilks’ Λ = 0.99, *F*(2,219) = 0.77, n.s.]. These results show that whereas participants were more dishonest in work session 2, our experimental conditions did not induce such dishonesty.

#### Goal difficulty

Our results show non-significant mean differences in goal difficulty between the goal-setting types across task types during work sessions 1 and 2. More precisely, our multivariate results show that mean differences across goal-setting conditions were non-significant for the time required to complete each task, regardless of the work session and the task type [Wilks’ Λ = 0.97, *F*(4,420) = 1.69, n.s., partial η^2^ = 0.12]. The results are available from the first author.

### Hypothesis testing

Box’s *M* test was non-significant for our RM-MANCOVA [*F*(105,66641.54) = 1.10, n.s.], suggesting that our multivariate results are trustworthy. Our between-subject results show a main effect of followers’ attribution of leader’s charisma [Wilks’ Λ = 0.88, *F*(3,202) = 9.97, *p* < 0.0001, partial η^2^ = 0.13] and of employment status [Wilks’ Λ = 0.96, *F*(3,201) = 3.05, *p* < 0.0001, partial η^2^ = 0.04]. Instead, perceived task complexity did not have an effect. Similarly, neither our goal-setting type nor leadership style manipulation had a main effect on goal attainment, goal commitment, and perceived task effectiveness. However, our results show a significant interaction effect between leadership styles and goal setting types [Wilks’ Λ = 0.93, *F*(6,202) = 2.51, *p* < 0.05, partial η^2^ = 0.04] and a between-within subjects’ interactive effect of time on leadership styles and goal setting types [Wilks’ Λ = 0.93, *F*(6,404) = 2.33, *p* < 0.05, partial η^2^ = 0.03]. Further, we found a between-within subjects’ interaction effect [Wilks’ Λ = 0.93, *F*(6,402) = 2.40, *p* < 0.05, partial η^2^ = 0.04], which shows that the observed mean differences across experimental conditions also differed across work sessions.

No main effects of goal setting type on our criteria were found in this sample. However, univariate analyses show a small to medium (*f* = 0.14) effect size of leadership style [*F*(1,203) = 3.88, *p* < 0.05; partial η^2^ = 0.02] on goal attainment. Pairwise comparison (*I–J*) between the authentic (*I*, *M* = 3.62, SE = 0.11, 95% CI [3.41, 3.82]) and contingent-reward (*J*, *M* = 3.32, SE = 0.11, 95% CI [3.11, 3.53]) conditions show significant mean differences (*I–J* = 0.30, SE = 0.15, *p* < 0.05). Finally, single cell analyses confirmed that the incremental effect of authentic leadership on followers’ outcomes was significant mostly in the self-set goals conditions. These results do not support hypotheses 1a to 1c, support hypothesis 2a but not hypotheses 2b and 2c, and fully support hypotheses 3a, 3b, and 3c.

**FIGURE 1:**
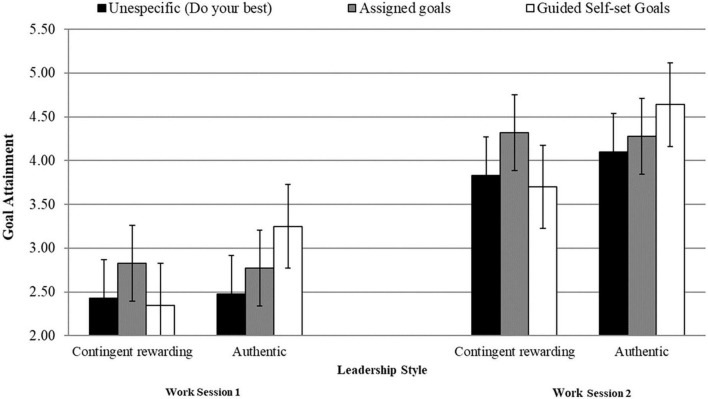
Interactive effects between goal setting types and leadership styles on goal attainment at work sessions 1 and 2.

**FIGURE 2:**
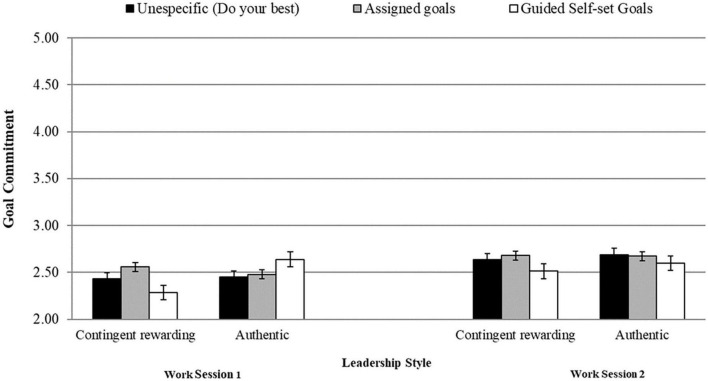
Interactive effects between goal setting types and leadership styles on goal commitment at work sessions 1 and 2.

**FIGURE 3:**
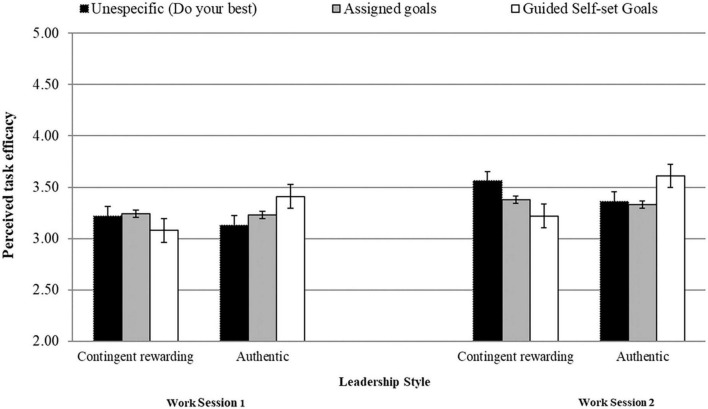
Interactive effects between goal setting types and leadership styles on perceived task efficacy levels at work sessions 1 and 2.

Table 1 displays means, standard deviations, and Pearson’s bivariate correlations. Given that the results of our RM-MANCOVA revealed a significant goal-setting type by leadership style by time interaction, Table 2 shows simple cell analyses for both work sessions. More precisely, in the guided self-set goals condition, goal attainment, goal commitment, and perceived task efficacy scores were significantly higher under an authentic leader than under a contingent-rewarding leader (see Table 2 and Figures 1–3). However, no significant differences existed between leadership styles for these criteria in the assigned-goals condition.

**TABLE 1:** Means, standard deviations, and bivariate correlations (*N* = 214).

	M	SD	1	2	3	4	5	6	7	8	9	10	11	12	13	14	15	16
1. DN	0.69	0.46	–															
2. TC – *T*_1_	3.48	0.75	0.13	–														
3. ALC – *T*_1_	3.06	0.78	0.02	0.10	–													
4. ES	0.32	0.47	−0.28**	−0.14*	–0.07	–												
5. LS	0.51	0.50	–0.02	–0.11	0.01	0.02	–											
6. AL – *T*_1_	3.14	0.57	0.07	0.04	0.53**	–0.12	0.05	–										
7. AL – *T*_2_	3.18	0.53	0.09	0.11	0.53**	–0.07	0.10	0.61**	–									
8. CR – *T*_1_	3.49	0.72	0.06	0.08*	0.55**	0.01	0.09	0.48**	0.44**	–								
9. CR – *T*_2_	3.61	0.64	0.02	0.10	0.41**	–0.12	0.08	0.34**	0.47**	0.51**	–							
10. DGS	0.34	0.47	–0.01	–0.02	–0.05	0.08	–0.05	–0.09	–0.06	–0.02	–0.05	–						
11. SSG	0.32	0.47	–0.06	0.07	0.04	0.05	0.05	–0.04	0.12	0.02	0.08	0.50**	–					
12. GC – *T*_1_	2.50	0.51	0.01	0.06	0.24**	–0.06	0.10	0.29**	0.25**	0.24**	0.17*	–0.01	–0.03	–				
13. GC – *T*_2_	2.64	0.46	–0.05	0.04	0.25**	0.07	0.03	0.19**	0.23**	0.13*	0.13	0.02	–0.10	0.50**	–			
14. GA – *T*_1_	2.73	1.25	0.04	–0.03	0.20**	0.05	0.08	0.24**	0.24**	0.37**	0.24*	0.03	0.12	0.33**	0.10	–		
15. GA – *T*_2_	4.23	1.27	0.07	–0.07	0.07	–0.06	0.13	0.12	0.17**	0.21**	0.18**	0.04	0.07	0.23**	0.13	0.63**	–	
16. PTE – *T*_1_	3.18	0.57	–0.05	0.03	0.22**	–0.12	0.06	0.30**	0.29*	0.30**	0.20**	–0.01	0.09	0.57**	0.31**	0.43**	0.37**	–
17. PTE – *T*_2_	3.43	0.58	–0.04	0.10	0.29**	–0.11	0.05	0.34**	0.34**	0.30**	0.33**	–0.05	0.01	0.43**	0.49**	0.25**	0.40**	0.58**

*p < 0.05; ** *p* < 0.01. DN, digital native (0 = No; 1 = Yes); task comp, perceived task complexity – T1 (manipulation check); ALC, followers attribution of charisma; ES, employment status (0 = No; 1 = Part time); LS, leadership style manipulation (0 = Contingent Rewards; 1 = Authentic); AL, perceived authenticity (manipulation check); CR, perceived contingent rewarding (manipulation check); DGS, assigned goals; SSG, self-set goals; GA, goal attainment; GC, goal commitment; PTE, perceived task efficacy, GA, goal attainment; T1, work session 1; T2, work session 2.

**TABLE 2:** Analyses of variances, means, standard deviations, *F*-values, and main effect sizes for goal setting types and leadership styles across cells.

	Unspecific goals (*N* = 72)		Assigned goals (*N* = 73)		Self-set goals (*N* = 68)	
			
	Work session 1	Work session 2	Work session 1	Work session 2	Work session 1	Work session 2
Goal attainment						
Contingent rewarding	2.46 (0.21)	3.86 (0.20)	2.87 (0.20)	4.36 (0.22)	2.52 (0.27)	3.70 (1.50)
Authentic	2.50 (0.21)	4.22 (0.20)	2.67 (0.21)	4.26 (0.23)	3.29 (0.20)	4.64 (1.08)
*F*-value	*F*(1,70) = 0.02, n.s.	*F*(1,70) = 1.60, n.s.	*F*(1,71) = 0.47, n.s.	*F*(1,71) = 0.09, n.s.	*F*(1,67) = 9.97**	*F*(1,67) = 9.70**
Cohen’s *f*	*f* = 0.00	*f* = 0.15	*f* = 0.08	*f* = 0.03	*f* = 0.32	*f* = 0.32
Goal commitment						
Contingent rewarding	2.51 (0.10)	2.67 (0.08)	2.53 (0.07)	2.66 (0.08)	2.28 (0.08)	2.53 (0.08)
Authentic	2.53 (0.10)	2.72 (0.08)	2.47 (0.07)	2.65 (0.08)	2.65 (0.08)	2.61 (0.07)
*F*-value	*F*(1,70) = 0.01, n.s.	*F*(1,70) = 0.15, n.s.	*F*(1,71) = 0.05, n.s.	*F*(1,71) = 0.02, n.s.	*F*(1,67) = 10.51**	*F*(1,67) = 0.50, n.s.
Cohen’s *f*	*f* = 0.00	*f* = 0.04	*f* = 0.09	*f* = 0.00	*f* = 0.40	*f* = 0.08
Perceived task efficacy						
Contingent rewarding	3.16 (0.10)	3.59 (0.11)	3.14 (0.09)	3.35 (0.09)	3.03 (0.64)	3.23 (0.09)
Authentic	3.08 (0.09)	3.34 (0.10)	3.19 (0.10)	3.42 (0.10)	3.32 (0.54)	3.61 (0.08)
*F*-value	*F*(1,70) = 0.31, n.s.	*F*(1,70) = 2.82^†^	*F*(1,75) = 0.18, n.s.	*F*(1,75) = 0.23, n.s.	*F*(1,67) = 3.12^†^	*F*(1,67) = 10.35***
Cohen’s *f*	*f* = 0.06	*f* = 0.20	*f* = 0.04	*f* = 0.00	*f* = 0.21	*f* = 0.39

^†^*p* < 0.10; **p < 0.01; ***p < 0.001. Bonferroni’s adjustment method was used to correct for multiple comparisons.

#### *Post hoc* analyses: Test of parallel vs. sequential mediation effects

Given that we did not detect the main effects of our independent variables on all of our outcome criteria, we conducted additional *post hoc* analyses to explore potential indirect effects. On the one hand, Latham et al. (2008) argue that goal commitment should be a mediator between goal setting, goal attainment, and perceived efficacy because “a person who is not committed to a goal by definition does not have one.” (p. 220). Instead, a potential counterfactual model would be that attaining goals at an earlier performance stage might increase goal commitment. In turn, goal commitment would increase how efficient individuals feel when performing later tasks, or what has been labeled the “high-performance cycle” by Latham et al. (2002).

For *post hoc* analyses, we used the PROCESS macro models 4 and 6 to construct three mediation model types (parallel vs. two sequential models). Further, we specified the bootstrapping of 10,000 subsamples to calculate SE and 95% CIs and activate the Bias-correction function. The PROCESS macro uses a Monte Carlo approach to bootstrap a certain number of sub-samples, deriving the SE and 95% CI empirically. Using bootstrapped CI to test mediation models can avoid issues introduced by asymmetric and other non-normal sampling distributions in the indirect effect residuals (Hayes, 2009). When bootstrapped 95% CIs do not include zero, statistical significance is achieved. Besides calculating bootstrapped SE and 95% CI, we required PROCESS to calculate Heteroskedastic consistent SEs (HC3) in our *post hoc* regression models (Hayes and Preacher, 2014). In this way, we report two sets of robust SEs derived from a theoretical (HC3) and empirical approach (Bootstrapped SE and CI).

Finally, we followed Cohen’s (1988) guidelines to calculate effect sizes and achieved statistical power (*f* for ANOVAs, *f*^2^ for regressions, and 1-β, respectively) with G*Power 3.1 (Faul et al., 2009). Finally, since version 3.3, the PROCESS macro enables incorporating multi-categorical variables as predictors in regression models, we entered our experimental conditions as predictors. The label “relative” indicates that the direct and indirect effects of any analysis using such a function will depend on how the independent variable is coded, even though the data being analyzed are otherwise the same (Hayes and Preacher, 2014).

**FIGURE 4:**
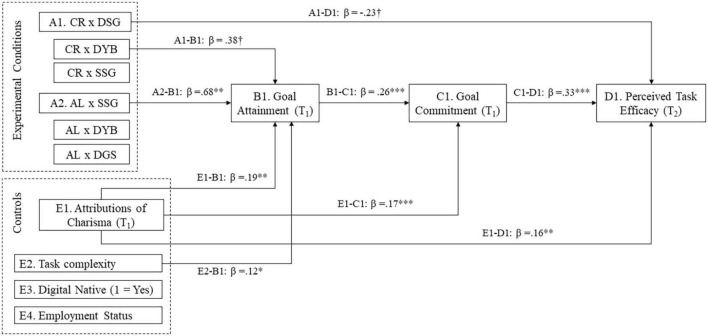
Sequential mediation model of the indirect joint effect of leadership styles and goal setting types on perceived task efficacy.

In line with prior results, when goal attainment and commitment were entered as parallel mediators, the 95% CI included zero. Similarly, in a model where goal commitment (T1) was the first stage mediator and goal attainment the second stage mediator (T1), the bootstrapped 95% CI for all relative indirect effects’ tests included zero. Instead, the bootstrapped 95% CI for a sequence mediation model where goal attainment is the first mediator (T1) and goal commitment the second mediator (T1) did not include zero only for the relative effect of authentic leadership and self-set goals condition (ES = 0.03, SE = 0.01, [0.01, 0.07]; see Table 3 and Figure 4).

**TABLE 3:** Multivariate regressions and bootstrapped 95% CI and SE for the indirect joint effect of leadership styles and goal setting types on perceived task efficacy, as sequentially mediated by goal attainment and goal commitment (*N* = 214).

	Goal attainment – T_1_			Goal commitment – T_1_			Perceived task efficacy – T_2_		
			
	β	Bootstrapped SE (HC3)	Bootstrapped 95% CI	β	Bootstrapped SE (HC3)	Bootstrapped 95% CI	β	Bootstrapped SE (HC3)	Bootstrapped 95% CI
First stage									
ALC – T_1_	0.18**	0.10 (0.10)	[0.10, 0.49]	0.17**	0.03 (0.03)	[0.01, 0.20]	0.16**	0.05 (0.05)	[0.02, 0.18]
TC – T_1_	0.12*	0.10 (0.11)	[0.02, 0.43]	0.11	0.05 (0.03)	[−0.01, 0.19]	0.10	0.05 (0.05)	[−0.02, 0.18]
DN	0.08	0.17 (18)	[−0.12, 0.54]	−0.01	0.07 (0.06)	[−0.14, 0.12]	−0.07	0.08 (0.08)	[−0.25, 0.07]
ES	0.07	0.18 (0.18)	[−0.16, 0.53]	−0.05	0.08 (0.07)	[−0.21, 0.11]	−0.08	0.08 (0.09)	[−0.26, 0.07]
CR × DYB (reference)	–	–		–	–	–	–	–	–
CR × DGS condition	0.38†	0.28 (0.29)	[−0.08, 1.02]	0.03	0.11 (0.11)	[−0.21, 0.22]	−0.36	0.13 (0.14)	[–0.47, 0.05]
CR × SSG condition	0.08	0.30 (0.31)	[−0.49, 0.69]	−0.40†	0.11 (0.12)	[−0.43, 0.01]	−0.40†	0.13 (0.15)	[–0.47, 0.03]
AL × DYB condition	0.10	0.29 (0.29)	[−0.42, 0.70]	0.11	0.14 (0.14)	[−0.20, 0.32]	−0.35	0.13 (0.15)	[–0.46, 0.07]
AL × DGS condition	0.22	0.28 (0.29)	[−0.28, 0.83]	−0.04	0.11 (0.11)	[−0.23, 0.19]	−0.21	0.12 (0.14)	[–0.35, 0.12]
AL × SSG condition	0.68**	0.28 (0.29)	[0.29, 1.38]	0.12	0.11 (0.11)	[−0.15, 0.27]	−0.11	0.12 (0.13)	[–0.29, 0.17]
	*R*^2^ = 0.11*	*f*^2^ = 0.12; 1-β = 0.96							
Second stage									
Goal attainment T_1_	–	–	–	0.28***	0.02 (0.03)	[0.06, 0.16]	0.12	0.03 (0.03)	[–0.01, 0.12]
				*R*^2^ = 0.18***	*f*^2^ = 0.21; 1-β = 0.999				
Third stage									
Goal commitment T_1_	–	–	–	–	–	–	0.30***	0.08 (0.10)	[0.19, 0.53]
							*R*^2^ = 0.28*	*f*^2^ = 0.39; 1-β = 0.999	

^†^*p* < 0.10; *p < 0.05; **p < 0.01; ***p < 0.001. T1, work session 1; T2, work session 2; ALC, attributions of leader charisma; DN, digital native (1 = Yes); ES, employment status; CR, contingent rewarding leadership; AL, leadership style manipulation (1 = Authentic); DGS, assigned goals; SSG, self-set goals; DYB, unspecific goals (“Do your best”). The CR × DYB condition is taken as reference category for out multi-categorical independent variable (Hayes and Preacher, 2014).

## Discussion

The main objective of the present study was to benchmark two sets of management practices in a controlled environment emulating a remote work setting. Our core prediction was that matching authentic leadership and followers’ self-set goals would result in higher goal attainment, stronger goal commitment, and higher perceived task efficacy than matching directive goal setting with contingent rewarding behaviors. Our data partially supported only one of six our main effects predictions (H2a) but fully supported our three interactive effects prediction (H3a, H3b, and H3c). In other words, our results suggest that the effects of specific managerial practices, such as goal setting types and leadership styles, are nuanced by the level of fit between them. Both traditional and positive managerial practices influence performance, but positive managerial practices have a stronger overall effect.

Further, in *post hoc* analyses, we explored indirect effects on our outcome criteria. More precisely, we compared if goal commitment and attainment work in parallel or a serial modality to influence perceived task efficacy. After testing three competing models, our *post hoc* analyses revealed that matching authentic leadership and self-set goals increased goal attainment (T1), which then increased goal commitment (T1) more than any other combination, even after controlling for attributions of charisma. In turn, goal commitment (T1) mediated the effect of goal attainment (T1) on participants’ overall perceived task efficacy (T2). In remote work contexts, a combination of positive management practices (self-set goals and authentic leadership) is a stronger driver of followers’ performance than a combination of traditional management practices (contingent reward and assigned goals) and even stronger driver than a mix between traditional and positive management practices (contingent rewards and self-set goals).

### Theoretical implications

Our work has implications for theory. First, our results substantiate our claim about how positive leadership can potentiate followers’ self-leadership, particularly for those employees working in remote settings. A detailed inspection of the recent empirical self-leadership studies reviewed shows that from all self-leadership behavior-focused strategies (Manz, 1986; Neck and Houghton, 2006), self goal-setting was the most consistent predictor of positive outcomes in remote work settings (Müller and Niessen, 2019; Costantini and Weintraub, 2022; Sjöblom et al., 2022). However, in our laboratory experiment emulating a remote work scenario study, we did not find a main effect of self-set goals on task performance-related outcomes. There are two competing explanations for this unexpected result, a counterfactual explanation, and our hypothesized interactive effect of set-set goals with a positive form of leadership.

The first (counterfactual) explanation is methodological. All the studies we reviewed employed endogenous, self-reported measures of goal setting types. Instead, our study employed an exogeneous behavioral measure goal setting types (Antonakis et al., 2010). Then, this methodological choice can behaviorally diminish the positive impact of the endogenous self-set goals on task performance. Further, by testing an interaction effect employing another exogenous independent variable (leadership style), we have confidence in the robustness of our results regarding the detected interaction effects.

Having discarded a potential methodological counterfactual explanation, we can support our theorizing about how matching a positive leadership style with a self-leadership behavioral strategy enhances the effect of self-set goals on task-related performance. Further, this enhancing effect cannot be explained by followers’ attributions of charisma eliciting “performance beyond expectations” (Bass, 1985), as we statistically isolated the expected effect of charisma, the most influential facet of transformational leadership (Bass, 1995). In other words, our results show that matching authentic leadership and set-set goals explains variance beyond followers’ attributions of charisma toward the leader.

Despite this encouraging finding, we acknowledge that we only tested one behavioral strategy of self-leadership. In our study, participants could not self-reward or self-punish themselves; rewards and punishments were administered by our “leader” within our pre-scripted user interface (see Supplementary Appendix III). Future studies could benefit from exploring how goal-setting types interact with other positive leadership styles beyond authentic leadership (e.g., servant, ethical, empowering, LMX, and so forth) and measuring more self-leadership behaviors.

Our work’s second theoretical contribution informs the study of positive leadership in remote work settings. We agree with Galanti et al. (2021) that “the COVID-19 outbreak has substantially forced most organizations to adopt this way of working, often without providing employees with the necessary skills required for remote work” (p. 426). However, in our laboratory experiment emulating remote work, we found a main, between-subjects effect of authentic leadership on participants’ goal attainment levels (H2a), even after controlling for remote followers’ attributions of charisma (regardless of the goal setting type condition).

Authentic leadership theory claims that exemplary role modeling is one mechanism by which authentic leaders elicit veritable and sustainable performance in followers (Avolio et al., 2004; Gardner et al., 2005). It would be a conceptual stretch to argue that our manipulation would trigger exemplary role modeling in a “face-to-face” context. However, we believe our manipulation might have been “good enough” to elicit exemplary role modeling in a remote work setting. In remote work settings, social interactions between agents are inherently mediated by some form of technology, also known as computer-mediated communication. Thus, in a computer-mediated communication context, we provided sufficient level of realism to trigger the effects of exemplary role modeling in our participants, even if purely in an episodic way.

A counterfactual explanation for such an increase in goal attainment could be that as participants interacted with our interface, these last might have developed dishonest “task-related strategies.” Dishonest “task-related strategies” while pursuing goals have been previously reported in goal-setting research. Such dishonest behaviors might arise when the goal commitment is high or when there are substantive implications for not reaching a performance goal (Schweitzer et al., 2004; Barsky, 2008; Ordóñez et al., 2009). However, in a heated academic exchange, Locke and Latham (2009) dismissed this argument, Schweitzer et al. (2004) claim was likely incidental and needed further replication.

In experimental studies, monetary incentives are the equivalent of the “high stakes” scenarios invoked in the research by Schweitzer et al. (2004) and Barsky (2008). Given that our experimental study did not include monetary incentives, this counterfactual explanation on dishonest behavior would not directly apply to our study. Indeed, participants in our study were incentivized with non-monetary reward releasing a motivational impact on decision making process (Thibault Landry et al., 2020). Furtherly, non-monetary incentives also remove self-selection bias in economic experiments (Abeler and Nosenzo, 2013).

Finally, our own results do not support the dishonesty counter-argument either. Although we detected some dishonest behaviors in our experimental tasks, said dishonest behaviors were distributed across experimental conditions, with no condition being significantly higher in dishonest behavior. Gerlach et al. (2019) found in a meta-analysis that the emergence of dishonest behaviors depend on both properties of the person and context, but such emergence also occurs in laboratory experiments. Thus, this counterfactual seems to lack merit to challenge our conclusions about the effect of authentic leadership on remote followers’ goal attainment.

### Practical implications

When ported to real-work settings, our results suggest that in the “new normal,” managers better motivate their remote employees by embracing positive management practices. Further, in real work settings, one could expect these practices to elicit positive employee attitudes and ideally trigger a virtuous spiral of individual performance. For example, one would expect positive management practices to increase the frequency of constructive trust episodes (Martínez-Tur and Peiró, 2009) and thus facility its emergence in remote work contexts (Jarvenpaa and Leidner, 2006; González-Navarro et al., 2010; Monzani et al., 2015b). Instead, our results suggest that trying to “monitor” performance from afar might be counter-productive for unleashing employees’ creative and analytical potential and actual capacities.

Finally, adopting the positive management practices explored in this study would take little to no investment other than training in positive management. Still, our results suggest that positive management could significantly increase employee performance in remote work settings. Instead, doing “business as usual” in a remote work setting will require investments in additional technology and risk having a counter-productive effect. By embracing positive management, leaders can help their firms to “do well by doing good” (Aguinis and Zedeck, 2011).

### Strengths, limitations, and future research directions

Our study has several strengths that ensure the robustness of our findings. First, we took great care to ensure that our design would not suffer from the traditional bias in social sciences, such as common method bias (Podsakoff et al., 2012) and endogeneity (Antonakis et al., 2010). We believe endogeneity should not be an issue in this study, as we manipulated our independent variables (goal-setting types and leadership styles). Further, we prevented common-method bias by combining them with behavioral outcomes (goal attainment) and self-reports (goal commitment and perceived task efficacy). Furthermore, the research team rated and adjusted goal attainment on generative tasks, a form of other task performance-ratings. Finally, we avoided issues of cross-sectional designs by conducting a panel experiment with two work sessions with multiple tasks in each experimental session.

As with any other study, our work is not without limitations that future research should address. First, there are some limitations in the chosen design of our study. In this regard, we acknowledge that two-thirds of our sample were unemployed students at the time of the experiment. We tried to mitigate this limitation by incorporating our participants’ employment status in our statistical analyses, thus statistically controlling for a potential variation that would influence our outcomes. For example, researchers might consider employing a larger number of work sessions and tasks. Thus, future studies should try replicating our results using additional data points. Replicating our findings in such an extended design would make a much stronger case for the temporal stability of our findings.

We also acknowledge as a design limitation that by conducting a laboratory experiment, we limited the ecological validity of our study. Whereas we tried to maximize the realism of our experimental setting and back story, the tasks were simplified for research purposes. Future studies should attempt to replicate our study by conducting a field experiment in a real-life work setting where participants can self-set goals when conducting more complex tasks.

Another related limitation is that we used a multimedia video instead of real-life actors for our leadership style manipulation. At first glance, the fact that our leader related to participants emulating a communication medium does not seem artificial, as video conferences in work meetings is becoming a cost-efficient alternative to traditional face-to-face meetings. Some may express concerns that our leadership manipulation was non-significant for women in work session 1. However, this finding aligns with prior results showing that male leaders displaying an authentic leadership style do not influence male and female followers equally (Eagly, 2005; Monzani et al., 2015, 2021).

Similarly, some may struggle with our decision to use only the contingent rewarding dimension of transactional leadership rather than the whole transactional leadership scale. Again, this choice is justified by our theorizing, as we needed a construct that best captured the nature of reinforcement-based styles. A better solution would have been to manipulate active management by exception while participants were performing. Such manipulation could be accomplished by having our leader actively interfere when participants deviate from their task parameters (e.g., repeating ideas in a generative task to increase their idea count). Unfortunately, this was beyond our design capabilities at the time of our experiment. Future studies should explore different leaders’ interventions while participants perform a task.

Third, participants in the guided self-set goals condition could not set less challenging task goals than those indicated by our software. Our rationale for this design choice was that providing the opportunity to set more, but not less, challenging goals signaled committed participants that they could adopt goal-related behaviors. Future research should improve our design by using a control condition with no goal-related manipulation of any kind.

## Conclusion

*“More than machinery we need humanity. More than cleverness we need kindness and gentleness.”*

*Charlie Chaplin*

The COVID-19 pandemic has changed how people work. In remote work contexts, such as work-from-home arrangements, engaging in traditional management practices such as directive goal setting and transactional exchanges might not be the best way to unleash employees’ potential. Instead, our study suggests that by embracing positive management practices, such as authentic leadership behaviors and allowing employees to self-regulate their work activities at home, managers can sustain or substantially increase their goal attainment, commitment, and perceived task efficacy.

## Data availability statement

The datasets presented in this study can be found in online repositories. The names of the repository/repositories and accession number(s) can be found in the article/Supplementary material.

## Ethics statement

The studies involving human participants were reviewed and approved by the Universidad de Valencia. The patients/participants provided their written informed consent to participate in this study.

## Author contributions

All authors listed have made a substantial, direct, and intellectual contribution to the work, and approved it for publication.

## Footnotes

1
https://www.pewresearch.org/internet/2021/02/18/experts-say-the-new-normal-in-2025-will-be-far-more-tech-driven-presenting-more-big-challenges/
