# Supplementary material for: Managing in the new normal: Positive management practices elicit higher goal attainment, goal commitment, and perceived task efficacy than traditional management practices in remote work settings. An experimental study
Source: Front Psychol. 2022 Oct 6;13:914616. doi: 10.3389/fpsyg.2022.914616 (PMC9586153; doi:10.3389/fpsyg.2022.914616)
Supplement: Supplementary file 1 [file Data_Sheet_1.docx]

Appendix I. *Experimental timeline for Work Session 1 and 2.*

| A. LS  manipulation | B. Task 1 | C. Task 2 | D. Task 3 | E. Task 4 | F. Task 5 | G. Task 6 | H. Post-session questionnaires | Time |
| --- | --- | --- | --- | --- | --- | --- | --- | --- |
| Multimedia video |  |  |  |  |  |  |  | 5’ |
|  | B.1 Task goal |  |  |  |  |  |  | 1.5’ |
|  | B.2 Generative 1 |  |  |  |  |  |  | 12’ |
|  | B3. Feedback | C.1 Task goal |  |  |  |  |  | 3’ |
|  |  | C.2 Intellective 1 |  |  |  |  |  | 8’ |
|  |  | C.3 Feedback | D.1 Task goal |  |  |  |  | 3’ |
|  |  |  | D.2 Generative 2 |  |  |  |  | 10’ |
|  |  |  | D.3 Feedback | E.1 Task goal |  |  |  | 3’ |
|  |  |  |  | E.2 Intellective 2 |  |  |  | 10’ |
|  |  |  |  | E.3 Feedback | F.1 Task Goal |  |  | 3’ |
|  |  |  |  |  | F.2 Generative 3 |  |  | 12’ |
|  |  |  |  |  | F.3 Feedback | G.1 Task Goal |  | 3’ |
|  |  |  |  |  |  | G.2 Intellective 3 |  | 8’ |
|  |  |  |  |  |  | G.3 Feedback |  | 3’ |
|  |  |  |  |  |  |  | H.1 Self-Report Measures | 8.5’ |
|  |  |  |  |  |  |  | Total Time | 90 min |

*Note: LS = Leadership style; Task goal = Task description and proximal goal-setting type manipulation; Feedback = Real time provision about goal attainment as shown in figure 2; Intellective = Single correct answer task; Generative = Idea generation task (Brainstorming). Both Work Sessions consisted of the same type of tasks and activities following the timeline shown above.*

Appendix II. Descriptions of task types for Work Sessions 1 and 2

|  | *Generative Tasks (e.g., brainstorming)* |  |  | *Intellective Tasks (e.g., single correct answer)* |
| --- | --- | --- | --- | --- |
| *Work session 1* | | | | |
|  | **1. Idea Trigger**: Generate possible sales arguments for a portable solar charger. |  |  | **1. Arithmetic problem**: Calculation of the amount of interest paid on a loan, based on a formula provided. |
|  | **2. Idea Trigger**: Provide reasons *against* downsizing and offshoring of the manufacturing department. |  |  | **2. Decision-making**: Choose among four final candidates for a position, based on CV and job description requirements. |
| 3 | **3. Idea Trigger:** Suggesting themes for an event in the renewable energy industry. |  |  | **3. Ranking:** Establishing an order for short and long-term quality improvement action controls when purchasing raw materials. (Participants had to rank 12 items using a drop-down menu: 6 short term and 6 long-term actions) |
| *Work session 2* | | | | |
| 1 | **1. Idea Trigger**: Suggesting Human Resources-related improvement actions to qualify for the “best place to work” award. |  | 2 | **1. Arithmetic problem:** Calculation of the amount of a purchase order for a product that has many components with different costs. |
| 3 | **2. Idea Trigger:** Provide reasons *in favor* downsizing and offshoring of the manufacturing department. |  | 4 | **2. Decision-making:** Selection of an optimal delivery route based on a series of simple criteria (the most cost-efficient route involved an unethical behavior, while the second-best option did not). |
| 5 | **3. Idea Trigger:** Generating ideas for a motivational speech based on the information provided by the CEO in the first work session. |  | 6 | **3. Ranking:** Rank 6 potential geographical expansion zones based on scores on multiple criteria shown in 6 bar charts. |

*Note: On all generative tasks, participants had to write ideas in a text box. On intellective tasks, participants had to write a single value or rank alternatives using a displayable menu.*

Appendix III. *Examples of Leadership style manipulations’ script.*

| *Work Session 1 – Multimedia Video* | | | | |
| --- | --- | --- | --- | --- |
|  | *Authentic leader* |  | *Contingent-rewarding leader* |  |
|  | ***Self-Awareness:*** “This role I am delegating to you implies a deep awareness of your strengths and weaknesses…I recommend that you try to evaluate your skills and abilities, so that you can do your best.”  ***Internalized moral perspective:*** “While our company is interested in maximizing profit, we are aware of our responsibilities in the way we generate those profits”.  ***Balanced processing of information:*** “After listening and talking with my early collaborators, I realized that I could not manage all areas of the organization by myself”.  ***Relational transparency:*** “I am an open person, and I like to speak frankly, telling things as they are. I also expect the same things from the members of this company.” |  | ***Contingent Rewards:*** “In the early days of this company, I told my early collaborators: If you accomplish the objectives we set today, tomorrow you will be part of the executive board. Nowadays, most of them are now part of the executive committee. This episode was revealing for me in terms of the management style this company requires: Clarifying roles and expectations and rewarding the performance of those who reach their goals.”  ***Active management by exception:*** “My best managers proactively face and solve problems, identifying and preventing them before a problem exists. They use their experience to keep unexpected events from altering the correct functioning and development of this company”. |  |
| *Work Session 2 – Multimedia Video* | | | | |
|  | ***Self-awareness:*** “Knowing our skills and abilities helps us to face problems with an informed and balanced perspective”  ***Moral perspective:*** “On some of the trials in the previous work session you were probably faced with moral dilemmas. I hope you behaved according to the value we expect to transmit in this company. We do not only care about profits, but also about how we attain them.” |  | ***Contingent rewarding:*** “I hope that in the previous work session you identified what can increase your performance and, consequently, your final score in this work session.”  ***Active management by exception:*** “In some of the trials of the previous work session you were probably faced with moral dilemmas. I hope you acted according to the values of our company: we exist to create and maintain profits for our shareholders. Any action that brings us closer to that goal is positive.” |  |
| *Example of Feedback after a generative task (Work session 2 - Task 5. Keynotes for a motivational speech)* | | | | |
|  | ***Successful goal attainment:*** Congratulations! You reached an adequate number of arguments to motivate our employees’ future performance. Of course, we will evaluate their content to see if they really transmit our company’s values and mission (e.g., using technology to improve society)  ***Failure to reach goal:*** I am sorry to inform you that the number of arguments you provided did not reach our expectations. Despite this, as we do in all generative task, we will evaluate whether the content reflects our company’s values and mission (e.g., using technology to improve society). |  | ***Successful goal attainment:*** Congratulations! With skills such as yours, employees will now understand the rewards that await them in this organization if they give their 110%!  ***Failure to reach goal:*** This was a good opportunity to show your skills, and you didn’t take advantage of it. To reach our strategic objectives, it is essential that our employees try to reach their maximum capacity. They will only do so if they receive solid arguments about the attractive rewards for their successful performance! |  |
